# The Effect of Dietary Supplements on Endurance Exercise Performance and Core Temperature in Hot Environments: A Meta-analysis and Meta-regression

**DOI:** 10.1007/s40279-021-01500-2

**Published:** 2021-06-15

**Authors:** Jennifer S. Peel, Melitta A. McNarry, Shane M. Heffernan, Venturino R. Nevola, Liam P. Kilduff, Mark Waldron

**Affiliations:** 1 A-STEM Centre, College of Engineering, Swansea University, Swansea, UK; 2 Defence Science and Technology Laboratory (Dstl), Fareham, Hampshire UK; 3 Welsh Institute of Performance Science, Swansea University, Swansea, UK; 4 School of Science and Technology, University of New England, Armidale, NSW Australia

## Abstract

**Background:**

The ergogenic effects of dietary supplements on endurance exercise performance are well-established; however, their efficacy in hot environmental conditions has not been systematically evaluated.

**Objectives:**

(1) To meta-analyse studies investigating the effects of selected dietary supplements on endurance performance and core temperature responses in the heat. Supplements were included if they were deemed to: (a) have a strong evidence base for ‘directly’ improving thermoneutral endurance performance, based on current position statements, or (b) have a proposed mechanism of action that related to modifiable factors associated with thermal balance. (2) To conduct meta-regressions to evaluate the moderating effect of selected variables on endurance performance and core temperature responses in the heat following dietary supplementation.

**Methods:**

A search was performed using various databases in May 2020. After screening, 25 peer-reviewed articles were identified for inclusion, across three separate meta-analyses: (1) exercise performance; (2) end core temperature; (3) submaximal core temperature. The moderating effect of several variables were assessed via sub-analysis and meta-regression.

**Results:**

Overall, dietary supplementation had a *trivial* significant positive effect on exercise performance (Hedges’ *g* = 0.18, 95% CI 0.007–0.352, *P* = 0.042), a *trivial* non-significant positive effect on submaximal core temperature (Hedges’ *g* = 0.18, 95% CI − 0.021 to 0.379, *P* = 0.080) and a *small* non-significant positive effect on end core temperature (Hedges’ *g* = 0.20, 95% CI − 0.041 to 0.439, *P* = 0.104) in the heat. There was a non-significant effect of individual supplements on exercise performance (*P* = 0.973) and submaximal core temperature (*P* = 0.599). However, end core temperature was significantly affected by supplement type (*P* = 0.003), which was attributable to caffeine’s *large* significant positive effect (*n* = 8; Hedges’ *g* = 0.82, 95% CI 0.433–1.202, *P* < 0.001) and taurine’s *medium* significant negative effect (*n* = 1; Hedges’ *g* = − 0.96, 95% CI − 1.855 to − 0.069, *P* = 0.035).

**Conclusion:**

Supplements such as caffeine and nitrates do not enhance endurance performance in the heat, with caffeine also increasing core temperature responses. Some amino acids might offer the greatest performance benefits in the heat. Exercising in the heat negatively affected the efficacy of many dietary supplements, indicating that further research is needed and current guidelines for performance in hot environments likely require revision.

## Key Points

**Table:** 

Exercising in the heat appears to affect the efficacy of many dietary supplements, suggesting that findings from research conducted on certain supplements in thermoneutral conditions are not necessarily transferrable to other environmental conditions.
Certain supplements, such as caffeine and nitrate, lack sufficient data to support their use as ergogenic aids in the heat, despite their efficacy in thermoneutral conditions, with caffeine also increasing core temperature responses. Some amino acids might offer the greatest performance benefits in the heat.
A potential risk is posed to those in physical performance domains (i.e., athletes or military personnel) due to the limited guidance on how to supplement appropriately for endurance exercise in hot environments.

## Introduction

The ergogenic effects of a number of dietary supplements on endurance exercise performance are well-established [1–5]. Indeed, recent position statements by the International Olympic Committee (IOC [6]), American College of Sports Medicine (ACSM [7]) and the Union of European Football Associations (UEFA [8]) provide specific recommendations for certain performance enhancing dietary supplements that are thought to have sufficient evidence for use by endurance athletes during training and competition. In tactical occupational settings, official legal information on the use of dietary supplements is often provided [9]; however, specific guidance on ergogenic aids is not. Despite this, the use of supplements among military personnel in training [10, 11] and during operations [12, 13] has been well reported. While it has been recognised that contextual factors should be considered when selecting dietary supplements [6, 9], there is limited guidance on this relating to endurance exercise performed in hot environments. This is particularly surprising, given that many endurance events and major international competitions take place in a combination of hot and humid conditions [14, 15]. For example, the forthcoming Tokyo 2021 Olympic Games are expected to take place in air temperatures exceeding 30 °C, with a humidity index of ~ 38 [16, 17]. Furthermore, military training and operations are also often conducted in extreme environments, in combination with prolonged endurance activity [18, 19].

Physical capacity is markedly impaired with increasing ambient temperature and humidity [20–23], leading to thermoregulatory strain and early onset fatigue, for a variety of physiological reasons [20, 24–32]. To perform optimally, environmental conditions—and their interaction with dietary supplement choices—must be carefully considered. Improper preparation for exercise in the heat can not only have detrimental effects on performance but can also lead to severe heat illness, and even death, in some extreme cases [33–36]. Therefore, a more comprehensive understanding of the effects of commonly used dietary supplements on physical performance and thermoregulation during exercise in the heat is necessary and could lead to safer and/or more efficacious heat preparation strategies.

The major limiting factors during exercise in the heat are linked to inexorable increases in core temperature [25], cardiovascular strain [29] and/or reductions in central drive [30]. Conceptually, regarding most endurance athletes and military personnel, the capacity to dissipate heat and offset one, or all, of these eventualities in hot environments predominantly occurs via three modifiable factors: lowered metabolic heat production, enhanced skin vasodilation (i.e., convective heat loss) or evaporative heat transfer (i.e., sweating [37–39]). The two supplements deemed to have the strongest empirical evidence to support these mechanisms [6], and reportedly serve to aid endurance exercise performance in temperate conditions, are caffeine (1,3,7-trimethylxanthine [1, 40]) and dietary nitrate (NO_3_^−^ [4]). Mechanistically, there is a sound theoretical basis for both caffeine and NO_3_^−^ supplementation to offset fatigue in the heat through increased central drive (caffeine [41]), and nitric oxide’s (NO) action on eccrine sweat gland function and subcutaneous microvascular control (NO_3_^−^ [42–44]). However, numerous studies have reported negative or null performance and thermoregulatory effects for both of these supplements during exercise in the heat [45–49].

The apparent failure of these well-evidenced supplements to produce an ergogenic effect in the heat is largely unexplained but could be due to the differing physiological demands of exercise in the heat, and a combination of factors limiting exercise tolerance in a hot environment [26–31]. It is also possible that ancillary physiological effects (i.e., on core temperature and blood pressure) of selected supplements have not been fully considered in accordance with environmental constraints and could inadvertently exacerbate symptoms of heat stress, which has been inferred from laboratory-based studies of caffeine [48] and NO_3_^−^ supplementation [50]. A similar line of reasoning can be applied to most other dietary supplements, based on the poor knowledge of their specific effects on thermoregulatory processes and subsequent ergogenic effects in the heat. Indeed, a number of alternative supplements have received some attention for their use in hot environments. For instance, supplementation with branched-chain amino acids (BCAAs [51]), tyrosine [52] and taurine [53] has been shown to extend time-to-exhaustion (TTE) in the heat, indicating that amino acids (AA) have ergogenic potential in hot conditions, yet these are not among those most commonly selected for training or competition purposes [54, 55]. Irrespective of the exact reasons for the apparent inconsistent findings within the published literature, there has not yet been a systematic evaluation of dietary supplements for endurance athletes and/or military personnel in the heat, which is necessary to clarify the most ergogenic options and those least likely to contribute to rises in core temperature.

Therefore, the aims of the current meta-analysis were to investigate the effects of selected dietary supplements on endurance performance in the heat, as well as the associated core temperature responses. The ergogenic effect of macronutrients [56–58] and eu/hyper-hydration [56, 59–64] on endurance exercise performance in the heat have been well-established and do not require revisiting here. However, the control of these factors among studies evaluating the efficacy of dietary supplements can be inconsistent, often precluding direct comparisons. Likewise, the training and acclimation status of participants has a significant effect on their thermoregulatory control and subsequent heat tolerance [65], as does the selected mode of exercise (i.e., time trial (TT) vs TTE [66]). This will affect behaviour and pacing during performance [67], yet these details appear to lack appropriate attention and have been largely overlooked in current consensus guidelines [6, 7]. Therefore, to understand the potential effects of dietary supplements on endurance performance in the heat, these factors were considered as potential moderating variables, forming part of the current meta-regression analysis.

## Methods

### Search Strategy

All literature that investigated the effects of dietary supplementation on exercise performance in a hot environment was searched and obtained using the Preferred Reporting Items for Systematic Reviews and Meta-analysis (PRISMA) guidelines, with a predetermined search strategy [68]. Medical subject headings (MeSH) terms were left active during the searches. There was no limit on the status, date or language of the publication. The single paper published in a language other than English was translated digitally using two separate translation software programs; Google Translate and DeepL Translator (DeepL GmbH, Cologne, Germany). The final Boolean searches were performed in PubMed and SPORTDiscus (EBSCO) on 6th May 2020. The search terms used were ‘(dietary supplements OR dietary supplementation OR nutritional supplements OR nutritional supplementation OR supplements OR supplementation OR ergogenic OR ergogenic aids OR caffeine OR creatine OR nitrate OR sodium bicarbonate OR beta-alanine) AND (heat OR cold OR temperature OR body temperature regulation)’ and all combinations were searched independently. The dietary supplements caffeine, creatine, nitrate, sodium bicarbonate and beta-alanine were searched for individually as they have been recognised by the IOC [6] and ACSM position statements [7] as having the greatest empirical evidence for their ergogenic effects in a thermoneutral environment and are, therefore, relevant to review in the heat. As there is no *a-priori* list of dietary supplements that are ergogenic through their effect on thermal balance, no other supplements were searched individually by name. All relevant supplements should be identified by the other search terms. Two authors (JP and MW) verified the search terms and the accuracy of the returned results.

### Study Selection

**Fig. 1:**
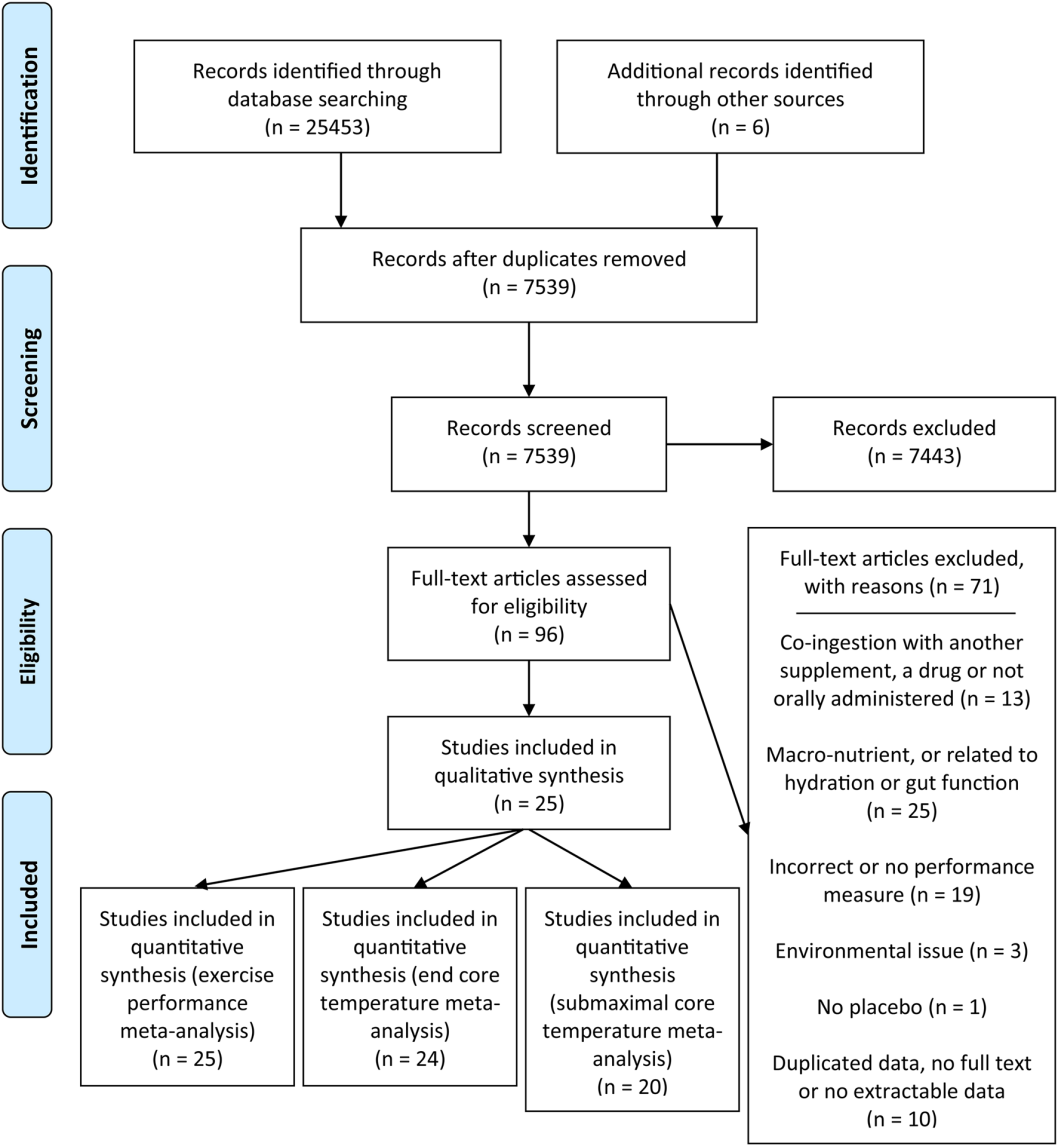
Process of study selection

Following the identification of all articles, the titles and abstracts were screened for inclusion by two reviewers and any duplicates removed. ‘Other sources’ were also identified, such as through social media (Twitter). The reference lists of the initial papers were reviewed independently by two authors (JP and MW). The remaining articles were then assessed separately (and without influence) by JP and MW against the inclusion and exclusion criteria. There was 100% agreement in study selection between the two reviewers. Papers were required to have been published in a peer-reviewed journal as original research articles with a cross-over, randomised controlled trial or an independent groups design. They must also have included a control or placebo group and participants were required to be healthy adults (≥ 18 years). To be included in this analysis, the studies must have passed through one of two filter points: they must have administered a dietary supplement (1) recognised by the IOC [6] and ACSM position statements [7] as having a strong evidence base for ‘direct’ improvements to performance; or (2) having a proposed ergogenic mechanism of action either directly or indirectly related to modifiable factors associated with thermal balance (i.e., skin blood flow, sweating, exercise efficiency). The studies must also have: (1) administered a dietary supplement (by our definition below); (2) evaluated exhaustive endurance exercise protocols performed for ≥ 75 s; and (3) been conducted in an ambient dry-bulb temperature of ≥ 30 °C in either a laboratory or field setting. Of the remaining papers, a number were further removed for the reasons outlined in Fig. 1. These largely comprised papers that included supplements that were: co-ingested; a drug; not orally administered; or a macro-nutrient (or had a mechanism of action which was considered to be directly related to hydration or gut function). Other reasons were the absence of a performance measure or one not adhering to the above definition; or environmental issues.

We defined a dietary supplement by adapting the IOC position statement [6]: a non-food, non-pharmacological, food component, nutrient or non-food compound that is purposefully orally ingested in addition to the habitual diet with the aim to ‘directly’ improve sports/exercise performance. The supplement is not being consumed for its indirect health benefits, its calorific value, its effects on hydration or gut function (the ergogenic mechanism of action is not through greater fluid absorption in the gut or increased gut permeability). The supplement is also legal as per the Misuse of Drugs Act 1971 [69] and is not on the World Anti-Doping Association’s prohibited substances list [70]. ‘Direct’ supplements refer to those acutely enhancing performance but not solely via “effective training, better recovery from training sessions, optimising mass and body composition, or reducing risks of injury and illness”.

Endurance performance encompasses a variety of activities, and the current analysis allowed for three forms of exhaustive exercise of any mode; TTE, TT and power output during closed loop tasks (i.e., intermittent sprint tests [IST]). Overall effects (i.e., combinations of all modalities) were considered for the analysis. Any forms of exercise that were either not exhaustive or performed for < 75 s were removed. This was based on the knowledge that exercise performed for ≥ 75 s has predominant contributions from aerobic metabolism, even at maximal intensities and irrespective of ambient temperature [71–73].

### Data Extraction and Quality Assessment

Data were independently extracted on separate occasions by two authors (JP and MW) and entered into a custom-designed Microsoft Excel spreadsheet. Extracted data included: (1) characteristics of the sample (sex, age, health, training and heat acclimation/acclimatisation status); (2) study design; (3) supplement, dose and timing of intake; (4) food and fluid intake before and during exercise i.e., hydration status, food intake before exercise and fluid ingestion during exercise; (5) environmental conditions (temperature and humidity); (6) performance outcomes; (7) end and submaximal core temperature (rectal, gastrointestinal, oesophageal or tympanic); and (8) bias. Risk of bias was assessed independently by two authors (JP and MW) according to Cochrane collaboration guidelines [74]. Where details of the study were unclear, the authors of the relevant papers were consulted for specific information or to clarify the method that was used. There was 100% agreement between the authors concerning the outcome of this quality assurance procedure; hence, it was not considered necessary to include a third independent reviewer. Standardised mean difference (SMD) was used to compare the results between studies utilising different protocols and measures. There were three outcome measures for this meta-analysis: (1) exercise performance; (2) core body temperature reported at the end of the exercise protocol, hereafter referred to as ‘end core temperature’; and (3) core body temperature reported at the mid-point of the exercise protocol, hereafter referred to as ‘submaximal core temperature’.

### Statistical Analysis

Data analysis was performed by one author (JP). Data were extracted from the qualifying papers in the form of a mean, standard deviation (SD) and sample size (*n*) for the meta-analysis. Publicly available software (WebPlotDigitizer, Version 4.3 [75]) was used to extrapolate any unreported values from the figures to mean and SD data. Authors of the original research articles were contacted for any missing data; however, where these were not accessible, they were imputed using the sample pooled SD from similar included studies [76]. Pre-to-post change scores were not used for any analysis, owing to their inconsistent availability. However, both sub-maximal and maximal core body temperature measures were reported to evaluate potential differences across stages of the exercise trials.

Three meta-analyses were conducted, i.e., one for each outcome measure. These were performed in RStudio [77, 78] and included 25, 24 and 20 comparison groups, for the exercise performance, end core temperature and submaximal core temperature meta-analyses, respectively. Not all studies reported end and submaximal core temperature; hence, they were excluded from the analysis. All data were analysed with a random-effects model, with heterogeneity assessed using the *I*^2^ statistic. Outliers were detected using a function in RStudio and influence on analysis investigated. Publication bias was accounted for by funnel plots and conducting Egger’s test and subsequently Duval and Tweedie’s trim and fill procedure, when indicated [79]. Hedges’ *g* and 95% confidence intervals (CI) were used to express SMD between dietary supplementation and placebo groups across studies. Sub-analysis of the different supplements included, and of the different exercise modalities utilised, were conducted for all three meta-analyses. Meta-regressions were also conducted to determine the effect of candidate moderators on exercise performance and core temperature outcomes, as reported in each study: training status (highly trained vs recreationally active); heat acclimation status (heat acclimated vs non-heat acclimated); hydration status (euhydrated vs hypohydrated); fluid ingestion during exercise (fluid ingestion vs no fluid ingestion); fasted vs fed state; exercise beforehand (exercise vs no exercise); heat exposure beforehand (heat exposure vs no heat exposure); duration of performance protocol; and total exercise duration. The thresholds for the magnitude of effects were < 0.2, 0.2, 0.5 and 0.8 for *trivial, small*, *medium* and *large* effects, respectively [80]. Alpha (*α*) was set at *P* ≤ 0.05 for all analyses.

## Results

### Study Selection

The initial searches retrieved 25,453 articles, plus one additional study through social media (Twitter). These were reduced to 7534 after removal of duplicates. After further screening and removal of reviews, animal studies and other irrelevant papers, 91 articles remained. Searches of the reference lists within those 91 reported studies provided five further papers. Of the 96 articles, 61 were removed based on the inclusion criteria and a further 10 were removed due to having: duplicate data with another paper, no full-text or no extractable data. This left 25 papers, of which 25, 24 and 20 papers were included in the exercise performance, end core temperature and submaximal core temperature analyses, respectively (Fig. 1).

### Study Characteristics

The characteristics of the 25 included studies are summarised in Table 1. The studies included a total of 272 participants, comprising both males and females (males 88%; both males and females 12%) of varying training (highly trained 56%; recreationally active 44%) and heat acclimation statuses (heat acclimated 16%; non-heated acclimated 56%; unreported 28%). Twenty-four of the studies had cross-over designs, while one study had an independent groups design (Table 1). Nine different types of supplements were included (caffeine, creatine, nitrate/beetroot [NO_3_^−^], BCAAs, tyrosine, vitamin E, Eurycoma longifolia Jack, taurine and polyphenols) in varying doses. These were a combination of single acute doses (*n* = 18; 72%) and chronic administration (*n* = 7; 28%). The performance measures included were TT (52%), TTE (44%) and IST (4%). The measures of core temperature were rectal (64%), tympanic (12%), oesophageal (4%), gastrointestinal (16%) and unreported (4%). Ambient temperature (mean 33.2 °C; range 30–42 °C), relative humidity (mean 47%; range 20–70%) and exercise time (mean 50 min; range 2–145 min) are reported herein. There were no adverse health-related events noted in any of the studies.

**Table 1:** Summary of studies included in the meta-analyses (*n* = 25)

Study	Design	Sample	Supplement, dose and timing	Temperature and relative humidity	Core temperature method	Exercise performance type	Outcome
Beaumont et al. [81]	Double-blind, randomised, repeated measures, cross-over	Healthy, recreationally active, non-heat acclimated males (*n* = 8). Age 22 ± 1 years	Caffeine 6 mg·kg^−1^ (60 min pre-exercise)	30 °C 50% RH	Gastrointestinal every 5 min (ECT + SCT)	60 min cycling @ 55% *W*_max_ followed by 30 min TT	NS ~ 3% ↑ in TT performance
Cheuvront et al. [45]	Double-blind, randomised, cross-over	Healthy, physically active, moderately fit, non-heat acclimated males (*n* = 10). Age 23 (18–37) years	Caffeine 9 mg·kg^−1^ (timing not mentioned)	40 °C 20–30% RH	Rectal every 5 min (ECT + SCT)	30 min cycling @ 50% $\dot{V}{\text{O}}_{{{\text{2peak}}}}$ followed by 15 min TT	NS ~ 2.4% ↑ in TT performance
Ferreira et al. [82]^a^	Double-blind, randomised, cross-over	Well-trained, heat acclimated, male cyclists (*n* = 8). Age 23.9 ± 8.6 years	Caffeine 5 mg·kg^−1^ (60 min pre-exercise)	30 °C average, ranged from 28.5–32 °C 71–78% RH	Tympanic pre and post exercise (ECT)	45 km cycling TT	NS ~ 4.2% ↑ in TT performance
Ganio et al. [83]	Double-blind, randomised, cross-over	Healthy, trained, non-heat acclimated male cyclists (*n* = 11). Age 25 ± 6 years	Caffeine 3 mg·kg^−1^ (60 min pre-exercise)	33 °C 41% RH	Rectal every 15 min (ECT + SCT)	90 min cycling @ 65% thermoneutral $\dot{V}{\text{O}}_{{{\text{2max}}}}$ followed by 15 min TT	NS ~ 6.3% ↑ in TT performance
Hanson et al. [48]	Single-blind, randomised, cross-over	Trained male (*n* = 6) and female (*n* = 4) endurance runners (n = 10). Age 26 ± 9 years	Caffeine 6 mg·kg^−1^ (60 min pre-exercise)	30.6 °C 50% RH	Gastrointestinal every 1 km (ECT + SCT)	10 km running TT	NS ~ 0.9% ↑ in TT performance
Ping et al. [84]^a^	Double-blind, randomised, cross-over	Recreational, heat acclimated male runners (*n* = 9). Age 25.4 ± 6.9 years	Caffeine 5 mg·kg^−1^ (60 min pre-exercise)	31 °C 70% RH	Rectal every 10 min (ECT)	Treadmill running @ 70% $\dot{V}{\text{O}}_{{{\text{2max}}}}$	Significant ~ 27.4% ↑ in TTE
Pitchford et al. [85]	Double-blind, randomised, counterbalanced, cross-over	Highly-trained, non-heat acclimated male cyclists (*n* = 9). Age range 22–42 years	Caffeine 3 mg·kg^−1^ (90 min pre-exercise)	35 °C 25% RH	Gastrointestinal continuously (ECT + SCT)	Total work cycling TT	NS ~ 6.9% ↑ in TT performance
Roelands et al. [46]	Double-blind, randomised, cross-over	Healthy, trained, non-heat acclimated males (*n* = 8). Age 23 ± 5 years	Caffeine 6 mg·kg^−1^ (60 min pre-exercise)	30 °C 50–60% RH	Rectal every 5 min (ECT + SCT)	60 min cycling @ 55% *W*_max_ followed by total work TT	NS ~ 3% ↓ in TT performance
Suvi et al. [86]^a,b^	Double-blind, randomised, cross-over	Healthy, physically active, non-heat acclimated males (*n* = 13) and females (*n* = 10; *n* = 23). Age 24.9 ± 4.1 vs 22.5 ± 2 years	Caffeine 6 mg·kg^−1^ (4 mg·kg^−1^ 60 min and 2 mg·kg^−1^ 0 min pre-exercise)	42 °C 20% RH	Measured but no extractable data	50 min treadmill walking @ 60% thermoneutral $\dot{V}{\text{O}}_{{{\text{2peak}}}}$ followed by TTE	NS ~ 4.3% ↓ in TTE
Kilduff et al. [87]	Double-blind, randomised, independent design	Endurance-trained, non-heat acclimated males (*n* = 11 vs 10; *n* = 21). Age 27 ± 5 vs 27 ± 4 years	Creatine 159.6 g (7 × 22.8 g·day^−1^)	30.3 °C 70% RH	Rectal every 5 min (ECT + SCT)	Cycling @ incremental work rate at 60–90 rpm	NS ~ 3% ↓ in TTE
Fowler et al. [49]	Double-blind, randomised, cross-over	Healthy, physically inactive, non-heat acclimated males (*n* = 11). Age 25 ± 5 years	Nitrate (NO_3_^−^) 46 mmol (5 × 9.2 mmol·day^−1^)	35 °C 28% RH	Rectal every 1 min (ECT + SCT)	Cycling @ thermoneutral gas exchange threshold at 70 rpm	NS ~ 9.7% ↑ in TTE
Kent et al. [88]	Double-blind, repeated measures, counter-balanced, cross-over	Endurance-trained male cyclists (*n* = 12). Age 26.6 ± 4.4 years	Nitrate (NO_3_^−^) 26 mmol (2 × 6.5 mmol·day^−1^ and 13 mmol 2 h pre-exercise)	35 °C 48% RH	Gastrointestinal every 20% work rate (ECT + SCT)	Total work cycling TT	NS ~ 3.1% ↑ in TT performance
McQuillan et al. [47]	Double-blind, randomised, cross-over	Healthy, well-trained endurance male cyclists (*n* = 8). Age 25 ± 8 years	Nitrate (NO_3_^−^) 24 mmol (2 × 8 mmol·d^−1^ and 8 mmol 90 min pre-exercise)	35 °C 60% RH	Rectal continuously (ECT + SCT)	20 min cycling @ 40–60% PPO followed by 4 km TT	NS ~ 0.3% ↑ in TT performance
Smith et al. [89]^a^	Double-blind, randomised, counterbalanced, cross-over	Recreationally-trained males (*n* = 12), Age 22 ± 4 years	Nitrate (NO_3_^−^) 6.2 mmol (3 h pre-exercise)	30 °C 70% RH	Tympanic post IST (ECT)	20 × 6 s sprints (114 s active recovery)	NS ~ 1.5% ↓ in mean power output
Cheuvront et al. [90]	Cross-over	Healthy, physically active, moderately fit, heat acclimated males (*n* = 7). Age 21 ± 2 years	BCAAs 14 g·kg^−1^ (0 min pre- and during exercise)	40 °C 20% RH	Rectal every 10 min (ECT + SCT)	60 min cycling @ 50% $\dot{V}{\text{O}}_{{{\text{2peak}}}}$ followed by 30 min TT	NS ~ 14.3% ↑ in TT performance
Mittleman et al. [51]	Double-blind, cross-over	Healthy, moderately-trained males (*n* = 7) and females (*n* = 6; *n* = 13). Age 24 ± 2.9 vs 25.6 ± 7 years	BCAAs Females (9.4 g) and males (15.8 g; 5 mL·kg^−1^ of 5.88 g·L^−1^ (Every 60 min at rest and 30 min during exercise)	34.4 °C 39% RH	Oesophageal every 5 min (ECT + SCT)	Cycling @ 40% $\dot{V}{\text{O}}_{{{\text{2peak}}}}$	Significant ~ 11.1% ↑ in TTE
Watson et al. [91]	Double-blind, randomised, cross-over	Healthy, endurance exercising, non-heat acclimated males (*n* = 8). Age 28.5 ± 8.2 years	BCAAs 4 × 250 ml at 12 g·L^−1^ (30 min intervals pre-exercise and 150 ml every 15 min during exercise)	30 °C 38% RH	Rectal every 10 min (ECT + SCT)	Cycling @ 50% $\dot{V}{\text{O}}_{{{\text{2peak}}}}$	NS ~ 6.6% ↑ in TTE
Coull et al. [92]	Double-blind, counter-balanced, cross-over	Recreationally active, non-heat acclimated males (*n* = 8). Age 23 ± 1 years	Tyrosine 150 mg·kg^−1^ (60 min pre-exercise)	40 °C 30% RH	Rectal every 5 min (ECT + SCT)	60 min treadmill walk followed by 2.4 km TT wearing a 25 kg backpack	NS ~ 5% ↑ in TT performance
Tumilty et al. [52]	Double-blind, randomised, cross-over	Healthy, endurance exercising, non-heat acclimated males (*n* = 8). Age 32 ± 11 years	Tyrosine 150 mg·kg^−1^ (60 pre-exercise)	30 °C 60% RH	Rectal every 10 min (ECT + SCT)	Cycling @ 68% $\dot{V}{\text{O}}_{{{\text{2peak}}}}$	Significant ~ 14.8% ↑ in TTE
Tumilty et al. [93]	Double-blind, randomised, cross-over	Endurance exercising, non-heat acclimated males (*n* = 7). Age 20 (range 26) years	Tyrosine 150 mg·kg^−1^ (60 pre-exercise)	30 °C 60% RH	Rectal every 5 min (ECT + SCT)	60 min cycling @ 57% $\dot{V}{\text{O}}_{{{\text{2peak}}}}$ followed by total work TT	NS ~ 1.1% ↑ in TT performance
Watson et al. [94]	Randomised, counter-balanced, cross-over	Physically active, trained, non-heat acclimated males (*n* = 8). Age 23 ± 3 years	Tyrosine 150 mg·kg^−1^ (2 h, 1 h, and during)	30 °C 50% RH	Rectal every 5 min (ECT + SCT)	Cycling @ 70% $\dot{V}{\text{O}}_{{{\text{2peak}}}}$	NS ~ 2% ↓ in TTE
Keong et al. [95]	Double-blind, randomised, cross-over	Recreational, heat acclimated male athletes (*n* = 18). Age 24.9 ± 1.4 years	Vitamin E No dose stated (6 week pre-exercise)	31 °C 70% RH	Rectal every 10 min (ECT + SCT)	Treadmill running @ 70% $\dot{V}{\text{O}}_{{{\text{2max}}}}$	NS ~ 5.3% ↑ in TTE
Muhamad et al. [96]^a^	Double-blind, randomised, cross-over	Healthy, male recreational athletes (*n* = 12). Age 23.3 ± 3.7 years	E. longifolia Jack 1200 mg (7 × 150 mg·d^−1^ and 150 mg 60 min pre-exercise)	31 °C 70% RH	Tympanic every 10 min (ECT)	60 min treadmill running @ 60% $\dot{V}{\text{O}}_{{{\text{2max}}}}$ followed by 20 min TT	NS ~ 3.6% ↑ in TT performance
Page et al. [53]	Double-blind, randomised, cross-over	Healthy, non-heat acclimated males (*n* = 11). Age 23 ± 2 years	Taurine 50 mg·kg^−1^ (2 h pre-exercise)	35 °C 40% RH	Rectal every 1 min (ECT + SCT)	Cycling @ thermoneutral ventilatory threshold at 80 rpm	Significant ~ 11.5% ↑ in TTE
Trinity et al. [97]	Double-blind, randomised, cross-over	Healthy, well-trained male cyclists (*n* = 12). Age 26.8 ± 5 years	Polyphenols 25,200 ppm (7 × 3600-ppm·day^−1^)	31.5 °C 55% RH	Rectal continuously (ECT + SCT)	10 min cycling @ 60–70% $\dot{V}{\text{O}}_{{{\text{2max}}}}$ followed by cycling @ 100% $\dot{V}{\text{O}}_{{{\text{2max}}}}$	NS ~ 3.5% ↓ in TTE

*TT* time-trial, *TTE* time-to-exhaustion, *IST* intermittent sprint test, *NS* non-significant, *PPO* peak power output, *Wmax* maximal workload, *ECT* end core temperature, *SCT* submaximal core temperature, *RH* relative humidity, $\dot{V}{\text{O}}_{{{\text{2max}}}}$ maximal oxygen uptake, $\dot{V}{\text{O}}_{{{\text{2peak}}}}$ peak oxygen uptake, *BCAAs* branched-chain amino acids

^a^Not included in submaximal core temperature analysis

^b^Not included in end core temperature analysis. The table is a reflection of participant characteristics, as reported by the authors of the articles

### Meta-analysis

**Fig. 2:**
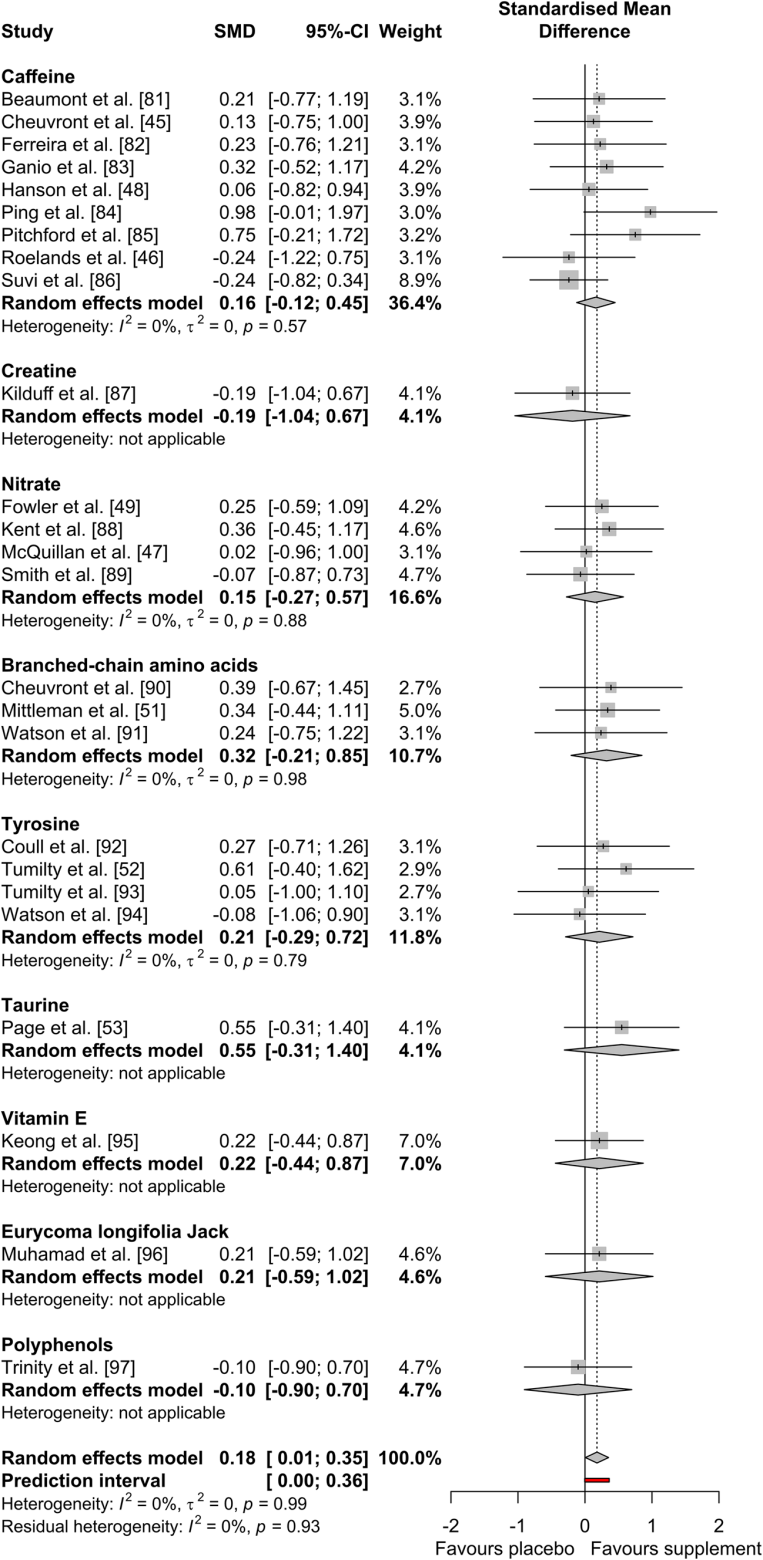
Effect of dietary supplementation on exercise performance

**Fig. 3:**
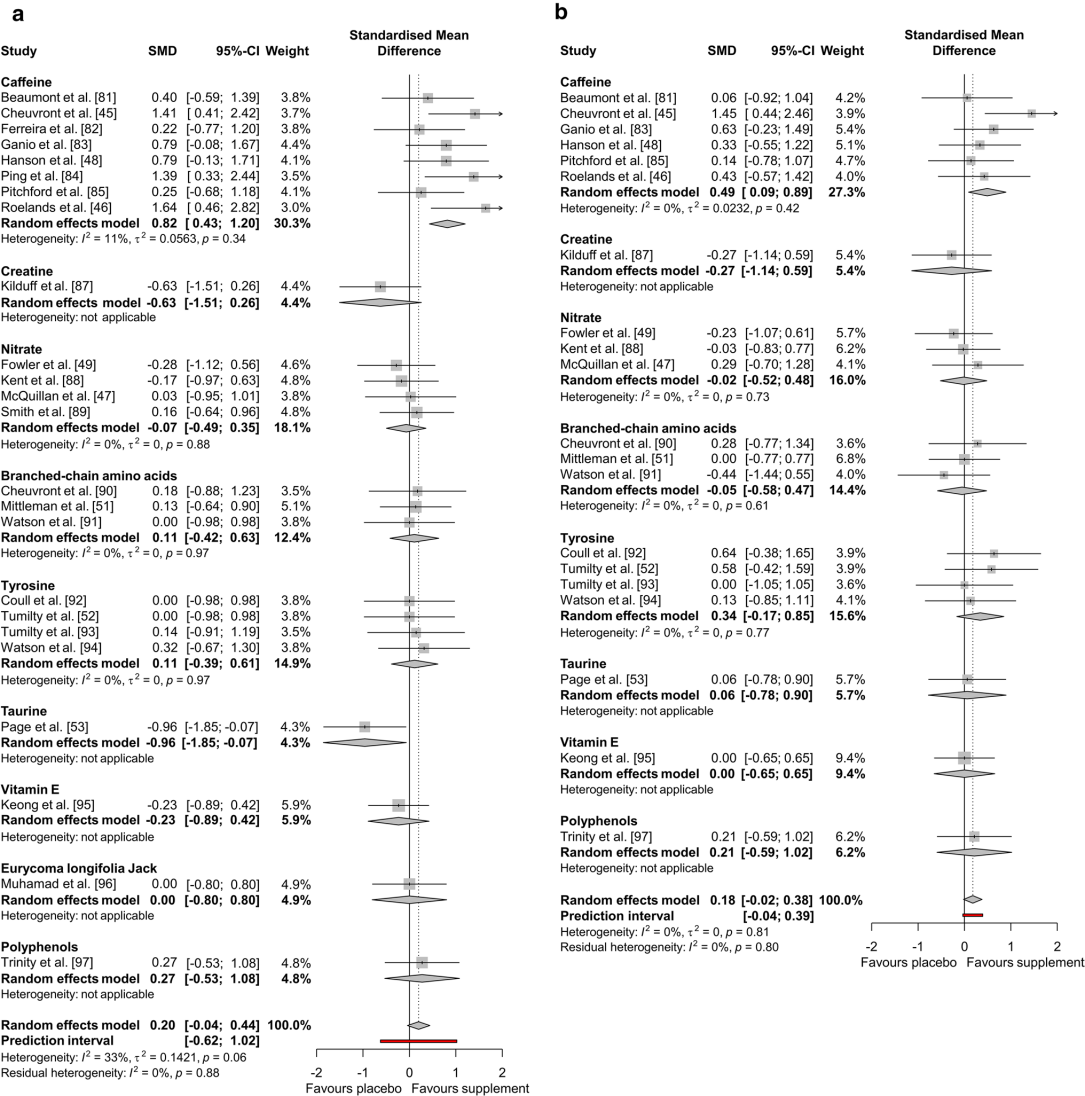
Effect of dietary supplementation on **a** end core temperature and **b** submaximal core temperature

The results of the performance meta-analysis (*n* = 25) are reported in Fig. 2. Overall, there was a *trivial* significant positive effect of all supplements on exercise performance compared to placebo (Hedges’ *g* = 0.18, 95% CI 0.007–0.352, *P* = 0.042). The *I*^2^ statistic demonstrated 0% heterogeneity. The results of the end core temperature (*n* = 24) and submaximal core temperature (*n* = 20) meta-analyses are reported in Fig. 3. Overall, end core temperature had a *small* non-significant increase (Hedges’ *g* = 0.20, 95% CI − 0.041 to 0.439, *P* = 0.104), and submaximal core temperature had a *trivial* non-significant increase (Hedges’ *g* = 0.18, 95% CI − 0.021 to 0.379, *P* = 0.080), with dietary supplementation compared to placebo, with 32.9% and 0% heterogeneity (*I*^2^), respectively.

### Sub-group Analysis

**Fig. 4:**
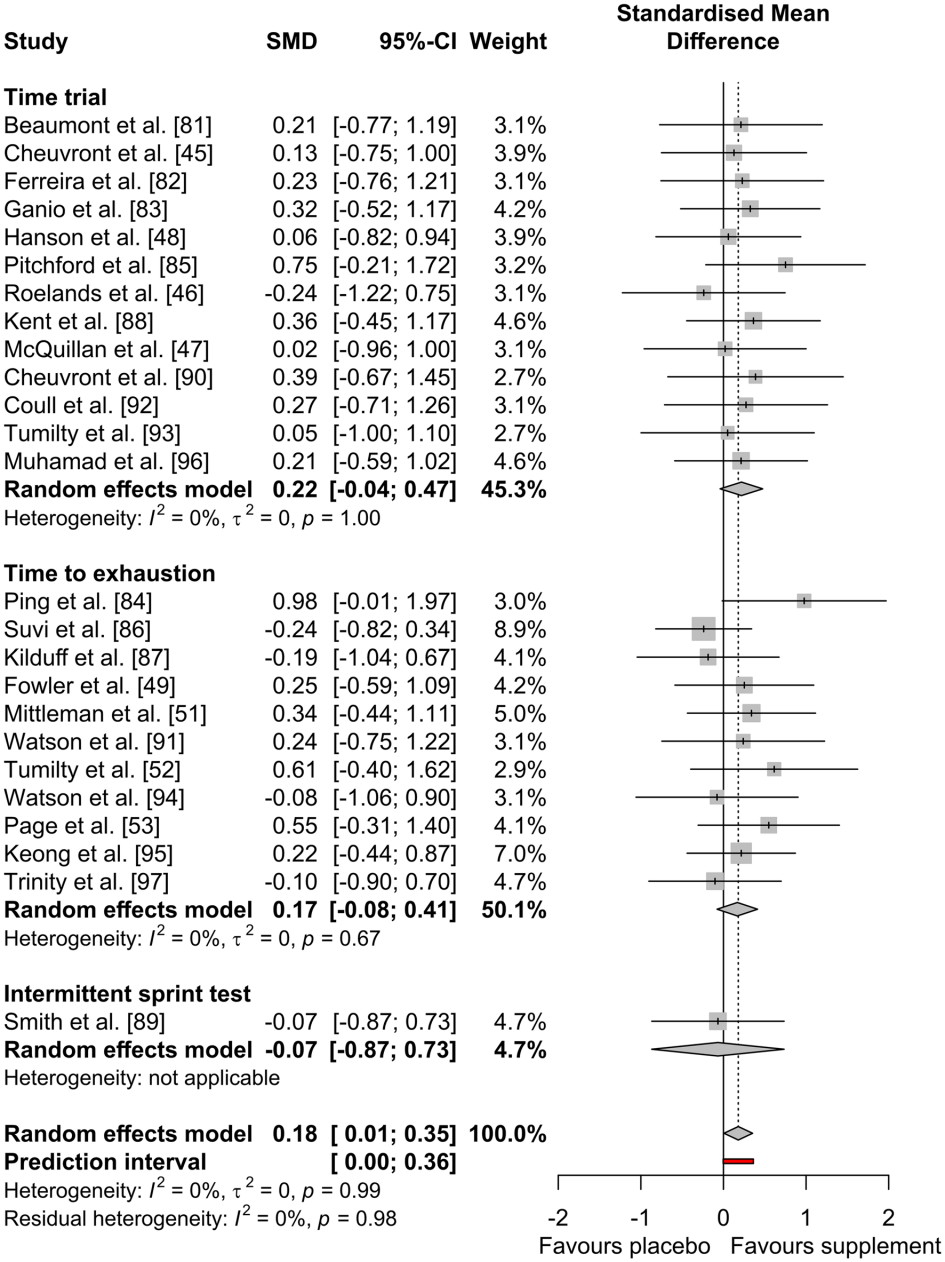
Effect of dietary supplementation on exercise performance by exercise modality

Sub-group analyses demonstrated a non-significant effect of the different supplement categories on exercise performance (*P* = 0.973). Caffeine (Hedges’ *g* = 0.16, 95% CI − 0.123 to 0.451, *P* = 0.263), creatine (Hedges’ *g* = − 0.19, 95% CI − 1.045 to 0.673, *P* = 0.671), nitrate (Hedges’ *g* = 0.15, 95% CI − 0.275 to 0.574, *P* = 0.490) and polyphenols (Hedges’ *g* = − 0.10, 95% CI − 0.903 to 0.698, *P* = 0.802) had a *trivial* non-significant effect. BCAAs (Hedges’ *g* = 0.32, 95% CI − 0.206 to 0.851, *P* = 0.232), tyrosine (Hedges’ *g* = 0.21, 95% CI − 0.288 to 0.717, *P* = 0.404), Eurycoma longifolia Jack (Hedges’ *g* = 0.21, 95% CI − 0.590 to 1.016, *P* = 0.603) and vitamin E (Hedges’ *g* = 0.22, 95% CI − 0.440 to 0.871, *P* = 0.520) had a *small* non-significant positive effect and taurine (Hedges’ *g* = 0.55, 95% CI − 0.306 to 1.403, *P* = 0.209) had a *medium* non-significant positive effect. Sub-group analysis of exercise modality (TTE, TT and IST) also demonstrated a non-significant effect of supplementation on exercise performance (*P* = 0.796). As shown in Fig. 4, the use of any supplement had a *trivial* non-significant effect on TTE (Hedges’ *g* = 0.17, 95% CI − 0.077 to 0.412, *P* = 0.178) and IST performance (Hedges’ *g* = − 0.07, 95% CI − 0.867 to 0.734, *P* = 0.870) and a *small* non-significant effect on TT performance (Hedges’ *g* = 0.22, 95% CI − 0.040 to 0.475, *P* = 0.097).

**Fig. 5:**
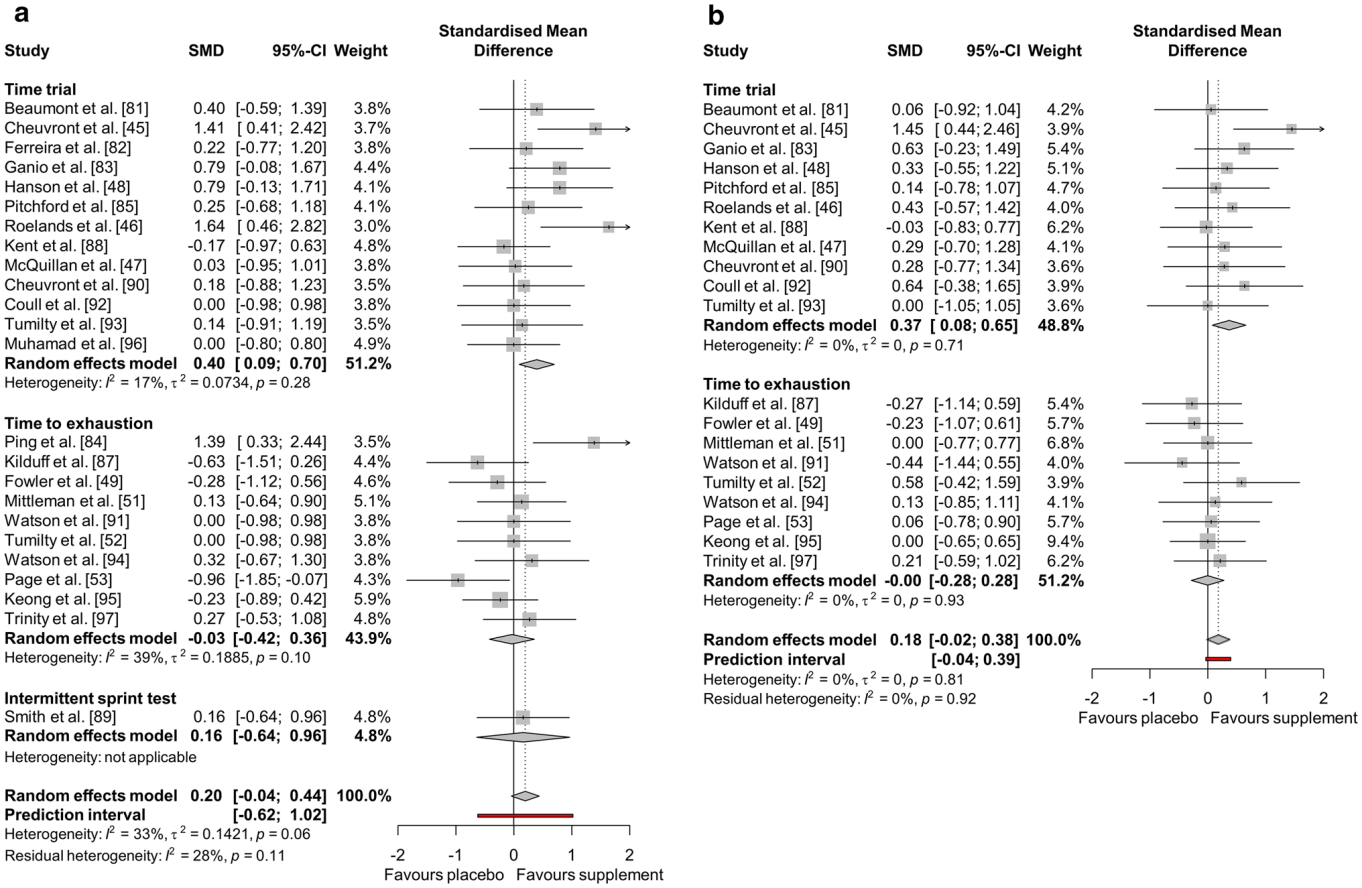
Effect of dietary supplementation on **a** end core temperature and **b** submaximal core temperature by exercise modality

Sub-group analysis demonstrated a significant effect of the different supplement categories on end core temperature (*P* = 0.003). Nitrate (Hedges’ *g* = − 0.07, 95% CI − 0.493 to 0.354, *P* = 0.748), BCAAs (Hedges’ *g* = 0.11, 95% CI − 0.418 to 0.631, *P* = 0.692), tyrosine (Hedges’ *g* = 0.11, 95% CI − 0.386 to 0.612, *P* = 0.658) and Eurycoma longifolia Jack (Hedges’ *g* = 0.00, 95% CI − 0.800 to 0.800, *P* = 1.000) had a *trivial* non-significant effect. Polyphenols (Hedges’ *g* = 0.27, 95% CI − 0.532 to 1.078, *P* = 0.506) had a *small* non-significant positive effect and caffeine (Hedges’ *g* = 0.82, 95% CI 0.433–1.202, *P* < 0.001) had a *large* significant positive effect. Vitamin E (Hedges’ *g* = − 0.23, 95% CI − 0.889 to 0.423, *P* = 0.487) had a *small* non-significant negative effect, creatine (Hedges’ *g* = − 0.63, 95% CI − 1.507 to 0.256, *P* = 0.164) had a *medium* non-significant negative effect and taurine (Hedges’ *g* = − 0.96, 95% CI − 1.855 to − 0.069, *P* = 0.035) had a *large* significant negative effect. Sub-group analysis of exercise modality demonstrated a non-significant effect of supplementation on end core temperature (*P* = 0.231). As shown in Fig. 5, the use of any supplement had a *trivial* non-significant effect on TTE (Hedges’ *g* = − 0.03, 95% CI − 0.417 to 0.355, *P* = 0.875) and IST (Hedges’ *g* = 0.16, 95% CI − 0.641 to 0.963, *P* = 0.694), but had a *small* significant positive effect on TT end core temperature (Hedges’ *g* = 0.40, 95% CI 0.093–0.699, *P* = 0.010).

Sub-group analysis demonstrated a non-significant effect of the different supplement categories on submaximal core temperature (*P* = 0.599). Nitrate (Hedges’ *g* = − 0.02, 95% CI − 0.517 to 0.482, *P* = 0.945), BCAAs (Hedges’ *g* = − 0.05, 95% CI − 0.580 to 0.474, *P* = 0.844), taurine (Hedges’ *g* = 0.06, 95% CI − 0.777 to 0.895, *P* = 0.890) and vitamin E (Hedges’ *g* = 0.00, 95% CI − 0.653 to 0.653, *P* = 1.000) had a *trivial* non-significant effect. Caffeine (Hedges’ *g* = 0.49, 95% CI 0.090–0.894, *P* = 0.016) had a *small* significant positive effect. Tyrosine (Hedges’ *g* = 0.34, 95% CI − 0.165 to 0.846, *P* = 0.187) and polyphenols (Hedges’ *g* = 0.21, 95% CI − 0.590 to 1.016, *P* = 0.603) had a *small* non-significant positive effect. Creatine (Hedges’ *g* = − 0.27, 95% CI − 1.136 to 0.586, *P* = 0.532) had a *small* non-significant negative effect. Sub-group analysis of exercise modality also demonstrated a non-significant effect of supplementation on submaximal core temperature (*P* = 0.070). As shown in Fig. 5, the use of any supplement had a *trivial* non-significant effect on TTE (Hedges’ *g* < 0.01, 95% CI − 0.281 to 0.278, *P* = 0.991), but had a *small* significant positive effect on TT submaximal core temperature (Hedges’ *g* = 0.37, 95% CI 0.082–0.654, *P* = 0.012).

### Meta-Regression

Across the three meta-analyses, there was only one moderating effect: that of exercise before the performance protocol (exercise vs no exercise) on submaximal core temperature responses (Table 2). Otherwise, there were no significant moderating effects of any variables on the outcome of exercise performance and end core temperature or submaximal core temperature responses (Table 2).

**Table 2:** Meta-regression outcomes

Moderator	Exercise performance	End core temperature response	Submaximal core temperature response
Training status	* β* = – 0.021, *P* = 0.907 (*n* = 25)	* β* = 0.095, *P* = 0.707 (*n* = 24)	* β* = – 0.084, *P* = 0.692 (*n* = 20)
Heat acclimation status	*β* = 0.247, *P* = 0.329 (*n* = 18)	*β* = 0.119, *P* = 0.770 (*n* = 17)	*β* = – 0.139, *P* = 0.660 (*n* = 15)
Hydration status	*β* = – 0.153, *P* = 0.783 (*n* = 16)	*β* = – 0.005, *P* = 0.994 (*n* = 16)	*β* = – 0.070, *P* = 0.909 (*n* = 12)
Fluid ingestion during exercise	*β* = 0.004, *P* = 0.983 (*n* = 22)	*β* = 0.222, *P* = 0.495 (*n* = 21)	*β* = – 0.082, *P* = 0.751 (*n* = 17)
Fed vs fasted state	*β* = 0.062, *P* = 0.763 (*n* = 19)	*β* = – 0.076, *P* = 0.819 (*n* = 18)	*β* = – 0.064, *P* = 0.793 (*n* = 15)
Acute heat exposure beforehand	*β* = – 0.144, *P* = 0.416 (*n* = 25)	*β* = 0.384, *P* = 0.113 (*n* = 24)	*β* = 0.363, *P* = 0.082 (*n* = 20)
Exercise beforehand	*β* = – 0.183, *P* = 0.312 (*n* = 25)	*β* = 0.421, *P* = 0.089 (*n* = 24)	*β* = 0.449, *P* = 0.039 (*n* = 20)
Duration of performance protocol	*β* = 0.002, *P* = 0.532 (*n* = 24)	*β* < 0.001, *P* = 0.919 (*n* = 23)	*β* = – 0.004, *P* = 0.152 (*n* = 19)
Total duration of exercise	*β* = 0.002, *P* = 0.491 (*n* = 24)	*β* = 0.004, *P* = 0.247 (*n* = 23)	*β* < 0.001, *P* = 0.952 (*n* = 19)

### Risk of Bias

**Fig. 6:**
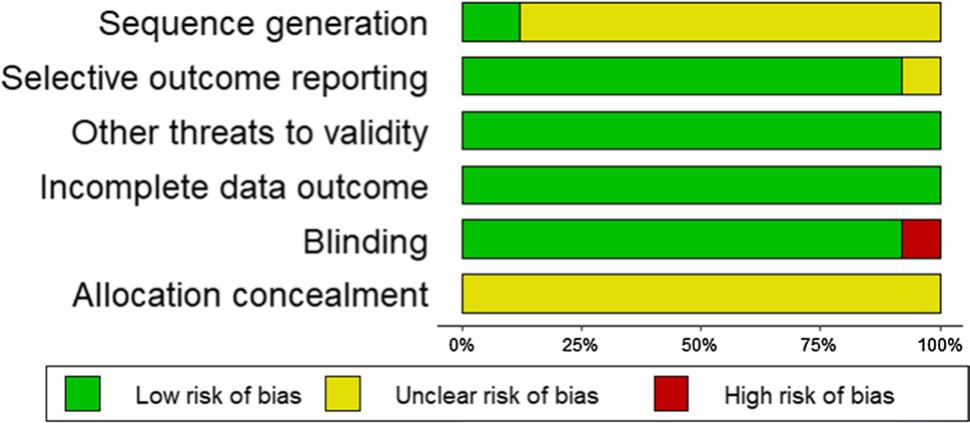
Risk of bias

**Fig. 7:**
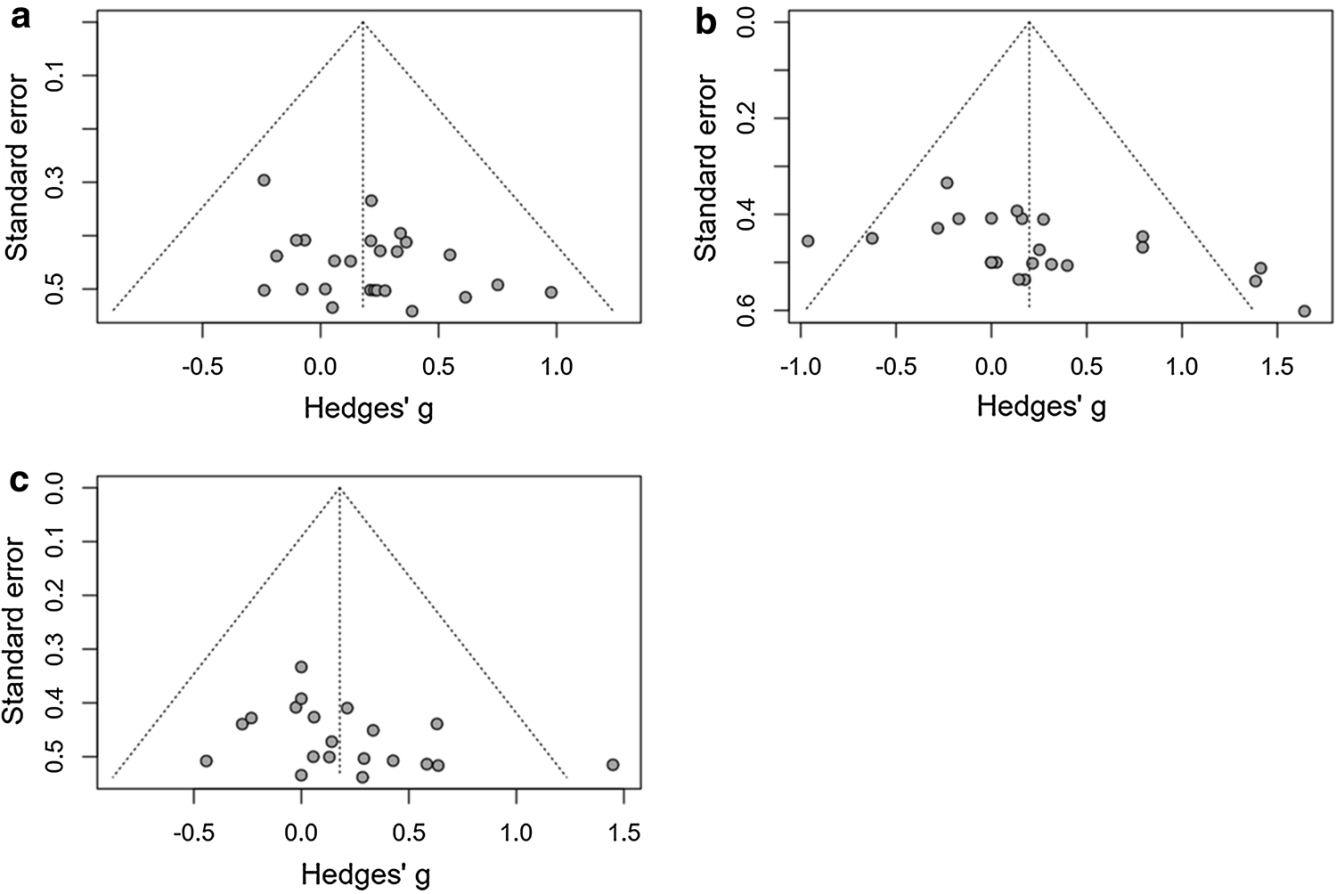
Publication bias for **a** exercise performance, **b** end core temperature and **c** submaximal core temperature

The studies included had a generally ‘low’ or ‘unclear’ risk of bias, with all but three studies not stating randomisation procedures [49, 53, 89], and two studies not adopting a blind design [90, 94]. Allocation concealment was ‘unclear’ in all studies (Fig. 6). There were no outliers detected and Egger’s test showed that there was no publication bias in the exercise performance meta-analysis (*P* = 0.053). Several outliers [46, 53] were detected in the end core temperature meta-analysis, owing to the large effects certain supplements appear to have on end core temperature responses. Egger’s test indicated publication bias (*P* = 0.015; Fig. 7), and therefore, Duval and Tweedie’s trim and fill procedure was conducted, but no meaningful adjustments to the data were made. One outlier was detected in the submaximal core temperature meta-analysis [45], but no publication bias was found (*P* = 0.115).

## Discussion

The main findings of the current meta-analyses were that dietary supplementation had a *trivial*, significant overall positive effect on endurance exercise performance in the heat (Hedges’ *g* = 0.18, *P* = 0.042; Fig. 2). The secondary sub-group analysis of exercise performance revealed no differences between supplements (*P* = 0.973); however, certain supplements, such as selected AAs, demonstrated the greatest performance effect sizes in this analysis. Of particular note, caffeine (Hedges’ *g* = 0.16, *P* = 0.263), creatine (Hedges’ *g* = − 0.19, *P* = 0.671) and NO_3_^−^ (Hedges’ *g* = 0.15, *P* = 0.490) had only a *trivial* and non-significant effect on endurance exercise performance in the heat, despite all of these supplements being recommended for athletes based on the strongest empirical evidence for performance enhancement in temperate conditions [6, 7]. The main findings of the core temperature analyses were that, overall, dietary supplementation had a *small* but non-significant positive effect on end core temperature (Hedges’ *g* = 0.20, *P* = 0.104), and a *trivial* non-significant effect on submaximal core temperature (Hedges’ *g* = 0.18, *P* = 0.080; Fig. 3). These results occurred irrespective of exercise duration, as demonstrated by the null effect of this moderating variable (Table 2). The secondary sub-group analysis of end core temperature demonstrated differences between supplements (*P* = 0.003), which was largely attributable to caffeine supplementation’s thermogenic effect. This evidence was surprising, given that some mechanisms underpinning the thermoneutral ergogenic effects of caffeine and NO_3_^−^, in particular, should, theoretically, facilitate thermal balance and performance in hot environments. These include lowered metabolic cost of exercise [98–100], peripheral vascular control (NO_3_^−^ [42–44]) and improved central drive (caffeine [41]). Therefore, the null findings presented herein have potentially profound implications for the use of these supplements in many performance scenarios, including major competitions or hazardous occupational settings. A possible explanation for this is that the effectiveness of otherwise established ergogenic dietary supplements is negated by the severity of hot environmental conditions. Regardless of the mechanistic reasons, these findings bring into question the depth of current understanding regarding supplementation in the heat and current recommendations should be tempered by this.

The analysis of core temperature revealed that caffeine had a *large* (Hedges’ *g* = 0.82, *P* < 0.001) and *small* (Hedges’ *g* = 0.49, *P* = 0.016) significant positive effect on end and submaximal core temperature, respectively. A significant rise in core temperature across exercise stages will deplete available heat storage capacity, leading to earlier onset of hyperthermic symptoms and reduced exercise performance [25]. This could explain the lack of an overall ergogenic effect for caffeine. Several papers have highlighted caffeine’s thermogenic effects [45, 46, 48], but none have directly linked this to negative performance outcomes. Therefore, the current meta-analytic approach was necessary to identify this important trend across studies. Caffeine’s effects are chiefly exerted via antagonism of centrally-located adenosine receptors, which act to increase the amount of circulating dopamine in the brain, as its release is inhibited by the binding of adenosine [41]. The inhibition of the reuptake of dopamine has been shown to increase core temperature [101], and therefore, a greater dopamine concentration in the brain following caffeine administration could explain the increase in core temperature demonstrated in the caffeine trials across studies. The oxygen uptake ($\dot{V}{\text{O}}_{{\text{2}}}$)response to exercise, at given exercise intensities, has also been reported to increase following caffeine ingestion compared to placebo, indicating increased metabolic heat production [102], which further supports this observation. Irrespective of any potential performance benefits, a supplement that increases core temperature when exercising in the heat could have potentially harmful effects. Given that heat illness during endurance events in hot environments is common and presents a risk to sports [34] or tactical athletes [33, 35, 36], such outcomes should be more clearly recognised in dietary guidance.

In the current meta-analysis, we also found a *trivial,* non-significant negative performance effect for polyphenols (Hedges’ *g* = − 0.10, *P* = 0.802), a supplement with known anti-oxidative properties. While a *small* positive effect was found for the other anti-oxidants, Eurycoma longifolia Jack (Hedges’ *g* = 0.21, *P* = 0.603) and vitamin E (Hedges’ *g* = 0.22, *P* = 0.520), there were no significant differences found herein or between the supplementation and placebo groups in the original research articles. Anti-oxidants are thought to delay fatigue by removing damaging reactive oxygen species (ROS) from the muscle and, therefore, counteracting exercise-induced oxidative stress [103]. It was somewhat unanticipated that anti-oxidants did not improve endurance exercise in the heat, since thermal stress exacerbates oxidative stress due to increased ROS production in such conditions [104]. A recent meta-analysis concluded that anti-oxidants have a moderate benefit to exercise performance in temperate conditions [105]; however, findings from individual studies remain equivocal. Studies reporting a considerable favourable effect on exercise performance administered a supra-physiological dose of *n*-acetylcysteine—a free radical scavenger—by intravenous infusion [106–108]. These findings are not supported by the majority of studies using oral anti-oxidant supplementation [109–118], with only a limited number finding a performance benefit [119–123]. It is possible that the dose and method of administration observed in the studies included in the current analysis were insufficient to elicit an ergogenic effect. In response to the current findings, further investigation into supplements conferring anti-oxidative effects in hot conditions is certainly warranted.

The supplements with the greatest ergogenic effect on exercise performance in the heat were AAs, with BCAAs (Hedges’ *g* = 0.32, *P* = 0.232) and tyrosine (Hedges’ *g* = 0.21, *P* = 0.404) having a *small* non-significant effect, and taurine (Hedges’ *g* = 0.55, *P* = 0.209) having a *medium* non-significant effect. While non-significant overall, the effects of AAs on exercise performance should not be discounted. Collectively, these supplements demonstrated the largest effect sizes, but there is currently insufficient evidence to recognise a significant effect. Interestingly, these are supplements with either equivocal or incomplete evidence for eliciting performance benefits in a thermoneutral environment [124–130]. The mechanism of action by which these AAs provide an ergogenic effect is not fully understood, but reduced central fatigue is commonly ascribed to the ergogenic effects of BCAAs and tyrosine [52, 131]. This theory suggests that a rise in plasma free fatty acid concentration due to prolonged exercise leads to tryptophan being displaced from albumin [132]. Consequently, the plasma concentration of unbound, free tryptophan increases, resulting in greater transport across the blood–brain barrier and subsequent synthesis of serotonin [131]. This, in turn, causes lethargy, loss of drive, reduced motor unit recruitment and, ultimately, fatigue [133, 134]. Amino acids, such as BCAAs and tyrosine are thought to compete with tryptophan for transport across the blood–brain barrier, thus limiting its entry into the central nervous system, reducing the rate of serotonin synthesis and delaying fatigue [135, 136]. Tyrosine is also a dopamine pre-cursor and dopamine plays a large role in increasing arousal, motivation and motor control [137]. Therefore, increased dopaminergic activity in the brain due to greater tyrosine concentrations may also delay fatigue, as well as increasing activation of motor pathways [138]. It is logical that these mechanisms could offset hyperthermic fatigue, as reduced central drive is observed during advanced heat stress, more so than during exercise in temperate conditions [31, 139]. However, while an overall positive effect of both BCAAs and tyrosine on performance within the current meta-analysis was demonstrated, the results of individual studies were inconsistent. The reasons for this are unclear, as while the exercise protocols, dosages (for BCAAs) and timings of ingestion differed slightly between studies, there were no apparent relationships between these variables and performance outcomes. Additional research is necessary to investigate this further.

Taurine, a sulphur containing AA, had the largest, albeit non-significant, effect on exercise performance in the heat of any of the supplements and also had a *large* significant negative effect on end core temperature (Hedges’ *g* = − 0.96 *P* = 0.035). This suggests that taurine exerts a thermoregulatory effect that reduces core temperature. Page et al. [53] demonstrated that taurine increased sweating onset and rate, which might explain the improved thermal balance. These effects, in combination with taurine’s capacity to enhance vasodilation [140], could facilitate both evaporative and dry heat transfer during exercise, delaying the rise in core temperature and hyperthermic fatigue. In the animal model, central infusion of taurine, a GABA agonist, has been shown to reduce core temperature in a dose-dependent manner [141]. Increased exogenous supply via oral supplementation could, therefore, offset the lower concentrations of GABA and taurine in hypothalamic nuclei following their heat stress-induced release [142, 143]. It should be stated that only one study [53] has been conducted regarding the effect of taurine supplementation on exercise performance in the heat, and therefore, further research needs to be conducted for corroboration and further mechanistic insight.

The secondary sub-group analysis of exercise modality (TT, TTE and IST) demonstrated no effect of supplementation on endurance exercise performance, or core temperature. However, dietary supplementation did affect TT performance end core temperature and submaximal core temperature. A possible explanation for this is that the TTs included in the current analysis were generally performed at higher intensities, which is likely to elicit greater metabolic heat production and subsequent core temperature responses. Only one of the meta-regression analyses performed was significant, where pre-trial exercise moderated (increased) the submaximal core temperature outcome. This was anticipated, because prior exercise may have already raised core body temperature to some degree, thus increasing submaximal core temperature. Collectively, these results indicate that the overall thermogenic effect of dietary supplements (driven largely by caffeine) could be exacerbated by performing TTs or by performing pre-trial exercise. This could be important for athletes performing in the heat, where TT race formats are common and are often preceded by a warm-up activity [144, 145]. Close monitoring of body temperature and other signs of heat strain might, therefore, be important if selected supplements are taken by athletes in hot TT races, alongside reduced intensity or duration of warm-up activities.

All candidate moderators, such as heat acclimation-, training-, hydration status, fluid ingestion during the trial and fed vs fasted state, did not affect exercise performance or core temperature responses to the supplements. For heat acclimation status and hydration status, this is likely due to the majority of papers mandating the recruitment of non-heat acclimated and hydrated participants. Mixed with the homogenously low effect found among most supplements in the heat, there was likely to be insufficient variation of data to establish a relationship between these variables and their effects. However, there was less consistent control of variables, such as training status, fluid ingestion during the trial, and fed vs fasted state, yet no moderating effect was found, indicating that these could not explain the variance found in any supplement's effect. On the basis of the current analysis, the effects reported could not be explained by candidate moderators but it would be useful to understand the efficacy of the most ergogenic supplements among participants of different training or acclimation statuses, given the effect of these processes on the acclimated phenotype [65] and the likelihood of this scenario in real-world athletic or occupational settings.

There are still a number of factors not fully investigated and which provide limitations to our current understanding of dietary supplementation for endurance exercise performance in the heat. The majority of papers used acute supplementation regimes, and therefore, the effect of chronic supplementation on exercise performance in the heat is still not well understood. Evaluation of this might be necessary for the more efficacious supplements observed here, such as taurine, and those with known benefits of chronic supplementation in thermoneutral conditions such as creatine, as this may elicit further effects. Similarly, the majority of exercise protocols were relatively short, with only nine trials exceeding 1 h, thus limiting the current understanding of certain supplements on prolonged exercise in the heat. This is particularly important, because prolonged exercise increases the probability of heat-related illness [146], which is extremely common in some occupations, such as military settings [147]. Finally, there was a lack of ‘real-world’ tasks performed in the studies included in the current meta-analysis, as all but one of the studies were controlled laboratory-based investigations. Therefore, the current results need replicating in ecologically valid conditions to establish their real-world effectiveness.

## Conclusion

In summary, for the first time, we have evaluated the effect of dietary supplementation on endurance exercise performance in the heat. Supplements such as caffeine and NO_3_^−^, which have the strongest empirical support for use in temperate conditions, lack sufficient data to support their use in the heat. Core temperature responses were also increased with caffeine supplementation, without any ergogenic benefit, which has potentially harmful health and performance consequences. Anti-oxidants also do not appear to provide a performance benefit in hot conditions. On the other hand, AAs appear to provide a greater performance benefit during exercise in the heat but the effects were often statistically insignificant. BCAAs offered the most consistent, yet *small*, performance effect, while taurine had both the greatest performance and thermoregulatory effect sizes of any of the supplements included in the current meta-analysis, albeit from a single study. Although further research is certainly needed, these supplements have potential to be effective for individuals exercising in hot environments. It appears that exercising in the heat significantly influences the efficacy of many dietary supplements, suggesting that findings from research conducted on certain supplements in thermoneutral conditions are not necessarily transferable to other environmental conditions. As such, research regarding the ergogenic effect of many dietary supplements for exercise in the heat is warranted. Future research should focus on understanding the mechanistic reasons for caffeine’s thermogenic effects and, conversely, the thermolytic effects of taurine. The inconsistent ergogenic effects of AAs also require further investigation, as the efficacy of their use is uncertain based on the current evidence. Collectively, our findings indicate that current dietary supplementation guidelines for exercise in hot environments must be adapted and require further detail for sports and tactical personnel.

## Declarations

### Availability of data and materials

Available on request.
